# Proportion of dementia in Australia explained by common modifiable risk factors

**DOI:** 10.1186/s13195-017-0238-x

**Published:** 2017-02-17

**Authors:** Kimberly Ashby-Mitchell, Richard Burns, Jonathan Shaw, Kaarin J. Anstey

**Affiliations:** 1 Centre for Research on Ageing, Health and Wellbeing, The Australian National University, Florey, Building 54, Mills Road, Acton, ACT 2601 Australia; 2 Baker IDI Heart and Diabetes Institute, 75 Commercial Road, Melbourne, VIC 3004 Australia

**Keywords:** Population attributable risk, Australia, Dementia

## Abstract

**Background:**

At present, dementia has no known cure. Interventions to delay onset and reduce prevalence of the disease are therefore focused on risk factor reduction. Previous population attributable risk estimates for western countries may have been underestimated as a result of the relatively low rates of midlife obesity and the lower weighting given to that variable in statistical models.

**Methods:**

Levin’s Attributable Risk which assumes independence of risk factors was used to calculate the proportion of dementia attributable to seven modifiable risk factors (midlife obesity, physical inactivity, smoking, low educational attainment, diabetes mellitus, midlife hypertension and depression) in Australia. Using a recently published modified formula and survey data from the Australia Diabetes, Obesity and Lifestyle Study, a more realistic population attributable risk estimate which accounts for non-independence of risk factors was calculated. Finally, the effect of a 5–20% reduction in each risk factor per decade on future dementia prevalence was computed.

**Results:**

Taking into consideration that risk factors do not operate independently, a more conservative estimate of 48.4% of dementia cases (117,294 of 242,500 cases) was found to be attributable to the seven modifiable lifestyle factors under study. We calculated that if each risk factor was to be reduced by 5%, 10%, 15% and 20% per decade, dementia prevalence would be reduced by between 1.6 and 7.2% in 2020, 3.3–14.9% in 2030, 4.9–22.8% in 2040 and 6.6–30.7% in 2050.

**Conclusion:**

Our largely theory-based findings suggest a strong case for greater investment in risk factor reduction programmes that target modifiable lifestyle factors, particularly increased engagement in physical activity. However, further data on risk factor treatment and dementia risk reduction from population-based studies are needed to investigate whether our estimates of potential dementia prevention are indeed realistic.

**Electronic supplementary material:**

The online version of this article (doi:10.1186/s13195-017-0238-x) contains supplementary material, which is available to authorized users.

## Background

Dementia describes a collection of symptoms associated with impaired memory and is characterised by progressive declines in thinking ability, physical function and behaviour [1]. Dementia has no known cure, and as it progresses so too does the inability to perform tasks of daily living [2]. At present, more than 40 million people worldwide are estimated to have the condition, with over US$600 billion spent on treatment and management [3, 4]. This figure is projected to increase to well over 70 million people by 2030 [4]. Such worrying future prevalence estimates highlight the need for urgent intervention focused on risk reduction because even a modest delay in onset can result in significant public health gains.

Cognitive decline and dementia are multi-causal. Research has shown that lifestyle factors (e.g. smoking habits, diet and physical inactivity) increase the risk of late-life dementia and that interventions targeting these can significantly reduce the population prevalence of dementia [5–7]. The potential impact of possible interventions to delay the onset of dementia on future prevalence of the condition has been reported previously for the world and specific regions [8, 9]. It is estimated that any intervention which could delay the onset of dementia by 1 year could reduce worldwide cases by 11% [10], while a 2-year and 5-year delay in onset could reduce the cumulative number of people developing dementia by 13% and 30% respectively [8]. In Australia, published research highlights that as little as a 10% reduction in dementia cases attributable to key modifiable lifestyle factors could result in savings of $280 million [11]. Delaying dementia onset therefore not only lessens the average number of years spent living with the disease but also has significant public health resource allocation implications [12].

Using a method published previously [9], we estimated the proportion of dementia in the Australian setting attributable to seven modifiable risk factors shown to be associated with the disease in the literature (midlife obesity, physical inactivity, smoking, low educational attainment, diabetes mellitus, midlife hypertension and depression).

Our study is novel because we take into account non-independence of risk factors, thereby providing more realistic population attributable risk (PAR) estimates for Australia than those obtained using the traditional Levin formula. In addition, we aimed to examine the effect of reducing the relative prevalence of each risk factor by 5%, 10%, 15% and 20% per decade (compounding reductions) on the future prevalence of dementia. Finally, we wanted to compare our PAR estimates with those produced previously for the USA, the UK, Europe and Australia.

To the authors’ knowledge, this is the only Australian study to provide estimates of PARs and future dementia prevalence taking into consideration non-independence of risk factors and utilising such a wide range of modifiable risk factors. This work will allow for comparison of Australia with other countries and other regions, and will inform dementia risk reduction policies.

## Method

PAR allows researchers and policymakers to estimate how much disease could be eliminated if there was a reduction in the prevalence of a causal factor or groups of interrelated factors [13]. For the calculation of PAR, relative risk and disease prevalence data are needed [14]. In the present study, the population prevalence of each risk factor was obtained from the 2011–2013 Australian Health Survey (the largest and most comprehensive health survey ever conducted in Australia) [15]. This survey represents a collation of the National Health Survey (*n* = 20,500 persons; one adult and one child from 15,500 households), the National Aboriginal and Torres Strait Islander Health Survey (*n* = 13,000), the National Nutrition and Physical Activity Survey (*n* = 12,000 persons; one adult and one child from 9500 households) and a National Health Measures Survey (11,000 survey participants aged 5+ years). The survey utilised a range of data collection methods including questionnaires, blood and urine tests and pedometers, and aimed to collect information about health status, risk factors, socio-economic circumstances, health-related actions, nutrition, physical activity and use of medical services. Table 1 presents the definitions for each of the risk factors included in the study.

**Table 1:** Risk factor definitions

Risk factor	Definition
Mid-life obesity	The proportion of adults (45–54 years) with BMI ≥ 30 (based on measured height and weight)
Physical inactivity	The proportion of adults not meeting physical activity guidelines based on self-reported physical activity engagement in the past 7 days and pedometer data (150–300 minutes of moderate intensity physical activity or 75–150 minutes of vigorous intensity physical activity, or an equivalent combination of both moderate and vigorous activities, each week)
Smoking	The proportion of adult smokers (based on self-report data)
Low educational attainment	The proportion of adults who have a primary and/or secondary school education (based on self-report data)
Diabetes mellitus	The prevalence of diagnosed diabetes mellitus among adults (based on self-report and biomedical data)
Midlife hypertension	The prevalence of hypertension in adults (aged 45–54 years) (based on self-report data)
Depression	Lifetime prevalence estimates of major depression using Diagnostic and Statistical Manual of Mental Disorders or International Classification of Diseases criteria (based on questionnaire data including diagnostic interviews and psychological distress scales)

Meta-analyses examining the association between dementia and the seven risk factors of interest were used to obtain relative risk data. Table 2 presents the relative risk and prevalence data utilised in this study and their sources.

**Table 2:** Prevalence and relative risk data sources

Risk factor	Prevalence	Relative risk	Communality (%)^a^
Midlife obesity	32.0^b^	1.64 (1.34–2.00)^d^	28.9
Physical inactivity	56.0^b^	1.39 (1.16–1.67)^e^	16.9
Smoking	16.1^b^	1.28 (0.99–1.60)^d^	11.3
Low educational attainment	24.0^b^	1.72 (1.52–1.96)^d^	12.9
Diabetes mellitus	5.4^b^	1.46 (1.20–1.77)^d^	30.6
Midlife hypertension	26.0^b^	1.61 (1.16–2.24)^f^	31.5
Depression	13.3^c^	1.65 (1.42–1.92)^d^	4.2

^a^ Estimated using the Australia Diabetes, Obesity and Lifestyle Study 2012 [32]

^b^ Obtained from ABS Health Survey 2012–2013 [29]

^c^ Obtained from AIHW, 2007 [30]

^d^ World Alzheimer Report, 2014 [21]

^e^ Obtained from Hamer et al., 2009 [31]

^f^ Obtained from Norton et al., 2014 [14]

### Statistical analysis

Levin’s Population Attributable Risk formula was used to calculate the proportion of dementia cases attributable to each of the risk factors under investigation [16]:

$$ \mathrm{P}\mathrm{A}\mathrm{R} = \kern0.5em \Big[ P\kern0.5em \times \kern0.5em \left( RR\kern0.5em -\kern0.5em 1\right)/\ \Big(1+ P \times \kern0.5em \left( RR\kern0.5em -\kern0.5em 1\Big]\right)\Big], $$

where *P* = population prevalence and *RR* = relative risk.

Still assuming independence of risk factors, we estimated their combined effect [17]:

$$ \begin{array}{l}\mathrm{Combined}\ \mathrm{PAR} = \kern0.5em 1\kern0.5em -\kern0.5em \left(1\kern0.5em -\kern0.5em  PA{R}_{midlife\  obesity}\right)\times \left(1\kern0.5em -\kern0.5em  PA{R}_{physical\  inactivity}\right)\times \\ {}\left(1\kern0.5em  - PA{R}_{smoking}\right)\times \left(1\kern0.5em -\kern0.5em  PA{R}_{low\  education\  attainment}\right) \times \left(1\kern0.5em -\kern0.5em  PA{R}_{diabetes\  mellitus}\right)\times \\ {}\left(1\kern0.5em -\kern0.5em  PA{R}_{midlife\  hypertension}\right)\times \left(1\kern0.5em -\kern0.5em  PA{R}_{depression}\right)\end{array} $$

We accounted for non-independence of risk factors by using a previously published modified formula which takes into account the unique contribution of each risk factor ‘*w*’ [9]:

$$ P A{R}_{Adjusted\  Combined}\kern0.5em =\kern0.5em 1\kern0.5em  - \Pi 1\kern0.5em -\kern0.5em \left( w \times \kern0.5em  PAR\right). $$

Factor analysis was used to estimate communality for each risk factor using data for adults aged 25 years and older from the Australia Diabetes, Obesity and Lifestyle Study—Wave 3 (AusDiab). The AusDiab is a population-based national survey of the general (non-institutionalised) Australian population aged 25 years and older.

The total number of dementia cases related to each of the seven risk factors was calculated as the product of their individual PARs and dementia prevalence.

The effect of reducing the relative prevalence of each risk factor by 5%, 10%, 15% or 20% per decade on the future prevalence of dementia in Australia was calculated using published dementia prevalence estimates for Australia [18, 19].

## Results

Table 3 presents the results of PAR calculations taking into consideration both independence and non-independence of risk factors. Confidence limits for PAR and the number of attributable cases were calculated using a published substitution method [20].

**Table 3:** PAR of dementia for each risk factor and number of cases attributable in 2010

Risk factor	Prevalence of risk factor	PAR % (95% CI)	Number of attributable cases in 2010 (95% CI)
Midlife obesity	32.0	17.0 (9.8–24.2)	41,222 (23,795–58,788)
Physical inactivity	56.0	17.9 (8.2–27.3)	43,468 (19,941–66,162)
Smoking	16.1	4.3 (–0.2 to 8.8)	10,460 (–391 to 21,362)
Low educational attainment	24.0	14.7 (11.1–18.7)	35,730 (26,906–45,410)
Diabetes mellitus	5.4	2.4 (1.1–4.0)	5878 (2591–9681)
Midlife hypertension	26.0	13.7 (4.0–24.4)	33,196 (9685–59,121)
Depression	13.3	8.0 (5.3–10.9)	19,296 (12,829–26,437)
Combined	–	57.0 (33.7–73.6)	138,020 (81,716–178,454)
Adjusted combined	–	48.4 (28.1–64.2)	117,294 (68,233–155,634)

Dementia cases 2010 = 242,500 [33]

*CI* confidence interval, *PAR* population attributable risk

Assuming independence of risk factors, we estimated that the seven risk factors examined contribute up to 57.0% of dementia cases in Australia. In order to account for interaction between risk factors, data for those aged 25 years and older from the AusDiab were used to estimate the shared variance for all seven risk factors (presented in Table 2). More specifically, to obtain communality we used STATA version 12 to generate a matrix of tetrachoric correlations and subsequently performed exploratory factor analysis using the correlation matrix as input. Similar to other studies, we used the Kaiser criterion for selecting the number of factors to retain [9]. Accounting for non-independence of risk factors, we estimated that the seven risk factors contributed 48.4% of dementia cases in Australia.

### Effect of risk factor reduction

**Fig. 1:**
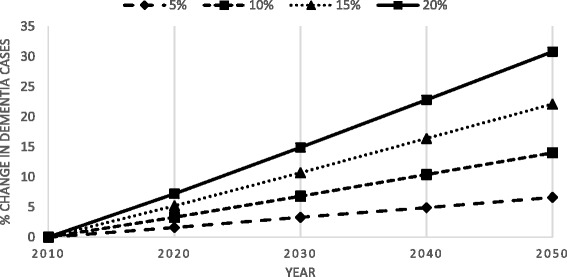
Percentage change in dementia cases as a result of a 5%, 10%, 15% and 20% reduction in each risk factor per decade. Estimated reduction in dementia prevalence that could result from a 5–20% per decade reduction in the prevalence of the risk factors under study

Table 4 and Fig. 1 show the effect of a 5%, 10%, 15% and 20% per decade reduction in each risk factor on future dementia prevalence estimates.

**Table 4:** Effect of a 5%, 10%, 15% and 20% per decade reduction in each risk factor on future dementia prevalence (2010–2050)

	Dementia estimate				
Reduction per decade	2010	2020	2030	2040	2050
	242,500	384,396	553,285	760,131	942,624
5%	242,500 (0.0%)	378,293 (1.6%)	535,831 (3.3%)	724,417 (4.9%)	884,021 (6.6%)
10%	242,500 (0.0%)	371,946 (3.3%)	517,987 (6.8%)	688,631 (10.4%)	826,632 (14.0%)
15%	242,500 (0.0%)	365,346 (5.2%)	499,850 (10.7%)	653,296 (16.4%)	771,870 (22.1%)
20%	242,500 (0.0%)	358,477 (7.2%)	481,528 (14.9%)	618,937 (22.8%)	720,965 (30.7%)

## Discussion

Assuming independence of risk factors, 57.0% of dementia cases in Australia could be related to the seven modifiable risk factors under study. Taking into consideration the non-independence of risk factors, we estimated that approximately 48.4% of dementia cases could be attributed to these risk factors. A reduction of between 5 and 20% per decade would have the effect of reducing future dementia prevalence by between 1.6 and 30.7% from 2020 to 2050. Our findings show that the percentage of cases explained by the risk factors under study is higher than that explained by APOE e4 (the main risk factor for AD). This highlights the importance of targeting modifiable risk factors in dementia reduction policies and programmes.

Similar to a previously published study which utilised data from the USA, Europe and the UK [14], we found that physical inactivity was related to the largest proportion of dementia cases. PAR estimates from our study and those published for the USA, Europe and the UK can also be compared because both studies examined midlife obesity, physical inactivity, smoking, low educational attainment, diabetes mellitus and midlife hypertension [9]. Although reported for AD cases, the authors of the international study suggest that their PAR estimates can be applied to the most common forms of dementia [9]. Overall, midlife obesity is related to a greater proportion of dementia cases in Australia (17.0%) than in the USA (7.3%), Europe (4.1%) and the UK (6.6%) [9], while physical inactivity is related to a relatively smaller proportion of cases in Australia (17.9%) than in the USA (21.0%), Europe (20.3%) and the UK (21.8%). Notably, a smaller proportion of dementia cases is attributable to smoking in Australia (8.7%) when compared with the USA (10.8%), Europe (13.6%) and the UK (10.6%). The proportion of dementia cases attributable to low educational attainment in the USA (7.3%), the UK (12.2%) and Europe (13.6%) is lower than that of Australia (14.7%). Our PAR estimate for diabetes mellitus is lower than those recorded for USA (4.5%) and Europe (3.1%) but higher than the UK estimate (1.9%). These differences may be predominantly due to the prevalence of these individual risk factors in each country and also to variations in risk factor definitions used between studies. For example, the midlife obesity prevalence for the USA utilised in Norton et al.’s study (13.1%) [14] is lower than our Australian estimate (32.0%) and also lower than the US Centers for Disease Control’s 2015 estimate (40.2%) [2]. The contribution of this risk factor to dementia prevalence is therefore likely to be significantly higher than calculated and more in line with our Australian PAR estimate. In addition, our estimate for low educational attainment may be higher than that published for other countries because our definition included all those who had a primary and/or secondary school education only (i.e. up to Year 12). Prior studies have used a less inclusive definition for low education (i.e. up to lower secondary schooling). We noted that the small difference between the analysis assuming independence and accounting for non-independence in our study (57.0% vs 48.4%) is in contrast to those published for the USA (52.7% vs 30.6%), Europe (54.0% vs 31.4%) and the UK (52.0% vs 30.0%). While we are unable to fully account for this observed difference, one possible explanation may be that it could be due to the effect of the computed weight ‘*w*’, which represents the proportion of the variance shared with other risk factors and which was comparatively higher in our study for all variables. While the assumptions on which the models for the UK, the USA and Australia are based are the same, the comparatively high ‘*w*’ value in Australia indicates that communality is low among risk factors and is suggestive of low co-morbidity among the risk factors under study. Further analysis of the primary dataset employed would need to be conducted in order to elucidate the exact cause of this disparity. It should, however, be acknowledged that low co-morbidity may be a result of various policy measures such as tobacco control efforts. Another important factor to be taken into account is the competing risk of death as a result of increased age and co-morbidities of older participants.

Our study builds on the methodology used in a recent Alzheimer’s Australia report based on 2014 ABS population projection data [11]. This report calculates PAR estimates assuming that risk factors operate independently. Here, we have accounted for non-independence of risk factors using a population-based sample and utilised compounding reductions each decade in order to determine the effect on future prevalence of disease. In both studies, however, physical activity explained the greatest proportion of dementia cases in the sample (24.8% and 17.9% respectively) [11]. A closer comparison reveals that the prior published estimates were higher for all commonly examined risk factors except midlife obesity, low education attainment and diabetes mellitus (midlife obesity: 13.9% vs 17.0%, physical inactivity: 24.8% vs 17.9%, smoking: 9.4% vs 4.3%, low educational attainment: 7.3% vs 14.7%, diabetes mellitus: 1.9% vs 2.4%, midlife hypertension: 16.3% vs 13.7% and depression: 8.9% vs 8.0%) [11]. These differences may be due to different sources of risk factor prevalence data and behaviour modification, for example smoking cessation. We were, however, unable to compare the combined effect of all examined risk factors because our study was the only one to take these into account.

Most of the effect size estimates used for relative risk in our study differ from those used in previous publications [9, 11]. While it would have been useful to utilise relative risks from Australian cohort studies, such data were not readily available for the risk factors being examined. As such, our relative risk estimates are taken from the World Alzheimer Report 2014 which included more recently published data [21]. The estimates used were lower for smoking and physical inactivity in our study but higher for midlife obesity and low educational attainment. Sensitivity analysis conducted using the relative risks used in Norton et al.’s study show that the proportions of dementia explained by midlife obesity (16.1%) and physical inactivity (31.5%) were higher in Australia than in the USA, Europe and the UK while that of smoking was lower (8.7%) (see Additional file 1). The Australian contribution of low educational attainment (12.4%) was higher than the USA and UK estimates but lower than that in Europe. PAR estimates for diabetes mellitus, midlife hypertension and depression remained unchanged because the same relative risks were used in both studies. Overall, both combined PAR and adjusted combined PAR were higher for our study when the relative risk estimates used in previous studies were utilised (Combined PAR:57.0% vs 64.3% and Adjusted Combined PAR: 48.4% vs 55.7% respectively).

Dementia may be delayed or prevented by targeting modifiable lifestyle factors [11, 22]. For example, Access Economics estimated that a 5-year delay in Alzheimer’s disease (the most common form of dementia) onset from 2005 would decrease prevalence by 48.5% in 2040 [12]. Other studies have calculated that any intervention which could delay onset by 5 years could decrease prevalence by between 37.0 and 44.0% [23, 24]. These projection estimates are higher than those reported in our study and notably do not take into account the dynamic interplay between risk factors which have been considered in our study. Although imprecise, our estimates are more realistic and conservative.

The modifiable lifestyle factors considered in our study have also been recognised as risk factors for developing other conditions such as cardiovascular disease and certain cancers, all of which are leading causes of death in Australia [25]. Our findings present a strong case for greater investment in lifestyle interventions in preventing dementia because these have the potential to reap other health and well-being benefits as well. Reducing the prevalence of or delaying the onset of dementia has the potential to lessen the impact of the disease, both financially and on individuals [26]. Delaying dementia onset lessens the average number of years spent living with the disease [12]. Those living with dementia for longer periods tend to require considerably more health services per annum than newly diagnosed individuals and this has substantial public health resource allocation implications [12].

Because physical inactivity was shown to contribute to the greatest proportion of dementia cases, this suggests that targeted interventions aimed at those who are not meeting recommendations may have the effect of reducing dementia prevalence. Policymakers, however, must be cognisant of the fact that no singular government intervention/policy, operating on its own, can directly reduce dementia onset/prevalence and change lifestyle habits [27]. Further research is needed to examine the monetary investment and time needed to reduce risk factor prevalence (especially physical inactivity prevalence) to a level that will result in significant improvement in the overall prevalence of dementia and other chronic diseases. It is also worth noting that while dementia risk reduction has the effect of increasing longevity and delaying onset of disease, it may not necessarily prevent the disease. Previous research has pointed to the longevity paradox—age is the strongest predictor of diseases that affect cognition and risk reduction has the potential to increase life expectancy [28].

To the authors’ knowledge, this is the only Australian study to provide estimates of population attributable risks (PARs) and future dementia prevalence taking into consideration non-independence of risk factors. This is also the only the Australian study to examine the contribution of such a wide range of modifiable risk factors. The methodology utilised in this article has only been reported on once in the literature and represents a notable addition to the traditional Levin formula used to calculate PAR in order to account for non-independence of risk factors [9]. In our calculations, we have utilised the most recent effect size estimates and have used a more realistic estimate of midlife obesity than other published work [14]. Our study therefore makes a valuable contribution to the research.

Limitations of using this method have been presented in a previous study [9]. These include use of biased adjusted relative risk estimates obtained from meta-analyses and the integrity of the method being untested [9]. However, although the methodology is still new, it is thought to provide a more realistic estimate of PAR. As further studies are conducted using this adjusted PAR calculation, there will be the opportunity to compare results across various geographic locations and test robustness. In interpreting our results, we indicate that it is beyond the scope of this study to prove that dementia can be prevented or that such major reductions in prevalence (up to 30.7%) are indeed possible. Further, the effects of risk factor reduction programmes depend on the stage of the lifecycle they are aimed to target, and many of our included risk factors focus on midlife. Further, there is a dynamic interplay among risk factors throughout the life course. In addition, it is possible that our figures overestimate the potential gain of risk reduction because they do not take into account that risk reduction leads to increased longevity which itself is a risk factor for dementia [28]. We are cognisant of our use of a relatively modest method and have considered utilising more informative models that examine the most likely future scenarios in our further work which take into account that each of the risk factors may be on a different trajectory. For example, educational attainment and smoking are both improving, while the prevalence of diabetes and obesity are increasing. Thus, improvements from the ‘base case’ are likely to be different for each risk factor. Further, as noted in a prior study, our analyses report on association between risk factors and disease and do not attempt to determine causality—the real link between the risk factors examined and dementia may be accounted for by other risk factors. Finally, while a substantial proportion of dementia cases was found to be attributable to the risk factors under study, we did not examine the contribution of other risk factors that have been examined in the literature for dementia such as fruit, vegetable, meat, fish and omega-3 intake and midlife serum cholesterol. Further studies are needed that take these into consideration. In addition, there is a need for more research into the effect of risk factor reduction on dementia from population-based studies in order to examine more nuanced issues such as effect size at various stages in the lifecycle and the costs and specific actions needed to have the greatest public health impact. Such data will no doubt prove useful to policymakers.

## Conclusion

Assuming that risk factors do not operate independently, approximately 48.4% of dementia cases in Australia can be potentially attributed to midlife obesity, physical inactivity, smoking and low educational attainment. Any intervention that reduces the prevalence of these by 5%, 10%, 15% or 20% per decade can have a significant public health impact, especially with regards to lowering the direct and indirect costs incurred by both governments and those living with the disease. Further research is needed which aims to provide policymakers with a set plan of action for achieving dementia risk reduction goals.

## Additional file

Additional file 1: Results of Sensitivity Analysis – PAR of dementia for each risk factor and number of cases attributable in 2010. Table showing the PAR estimates obtained for Australia if the relative risks used in the Norton et.al. 2014 paper had been utilised in the present study. (DOCX 14 kb)

## Abbreviations

ABS: Australian Bureau of Statistics
AD: Alzheimer’s disease
AusDiab: Australia Diabetes, Obesity and Lifestyle Study
PAR: Population attributable risk
RR: Relative risk
