# Supplementary material for: Proportion of dementia in Australia explained by common modifiable risk factors
Source: Alzheimers Res Ther. 2017 Feb 17;9:11. doi: 10.1186/s13195-017-0238-x (PMC5316209; doi:10.1186/s13195-017-0238-x)
Supplement: Additional file 1: — Results of Sensitivity Analysis – PAR of dementia for each risk factor and number of cases attributable in 2010. Table showing the PAR estimates obtained for Australia if the relative risks used in the Norton et.al. 2014 paper had been utilised in the present study. (DOCX 14 kb) [file 13195_2017_238_MOESM1_ESM.docx]

*Additional file 1: Table S1: Results of Sensitivity Analysis - PAR of dementia for each risk factor and number of cases attributable in 2010*

| Risk Factor | Prevalence of Risk Factor | RR used in our study | RR used in Norton et.al. study | PAR % (95% CI) obtained in our study | PAR % (95% CI) obtained in our study using RR estimates from Norton |
| --- | --- | --- | --- | --- | --- |
| Midlife obesity | 32.0 | 1.64 (1.34 – 2.00) | 1.60 (1.34 – 1.92) | 17.0 (9.8 – 24.2) | 16.1 (9.8 – 24.2) |
| Physical inactivity | 56.0 | 1.39 (1.16 – 1.67) | 1.82 (1.19 – 2.78) | 17.9 (8.2 – 27.3) | 31.5 (8.2 – 27.3) |
| Smoking | 16.1 | 1.28 (0.99 – 1.60) | 1.59 (1.15 – 2.20) | 4.3 (-0.2 – 8.8) | 8.7 (-0.2 – 8.8) |
| Low educational attainment | 24.0 | 1.72 (1.52 -1.96) | 1.59 (1.35 – 1.86) | 14.7 (11.1 – 18.7) | 12.4 (11.1 – 18.7) |
| Diabetes mellitus | 5.4 | 1.46 (1.20-1.77) | 1.46 (1.20-1.77) | 2.4 (1.1 – 4.0) | 2.4 (1.1 – 4.0) |
| Midlife hypertension | 26.0 | 1.61 (1.16 – 2.24) | 1.61 (1.16 – 2.24) | 13.7 (4.0 – 24.4) | 13.7 (4.0 – 24.4) |
| Depression | 13.3 | 1.65 (1.42 – 1.92) | 1.65 (1.42 – 1.92) | 8.0 (5.3 – 10.9) | 8.0 (5.3 – 10.9) |
| Combined | - | - | - | 57.0 (33.7 – 73.6) | 64.3 (33.7 – 73.6) |
| Adjusted combined | - | - | - | 48.4 (28.1 – 64.2) | 55.7 (28.1 – 64.2) |

*Note: Dementia cases 2010 = 242,500 (*[*26*](#_ENREF_26)*)*
